# Uniaxial films of maximally controllable response under visible light

**DOI:** 10.1038/s41598-020-69770-w

**Published:** 2020-08-03

**Authors:** Dias Tulegenov, Constantinos Valagiannopoulos

**Affiliations:** Department of Physics, Nazarbayev University, 010000 Nur-Sultan, Kazakhstan

**Keywords:** Optical materials and structures, Metamaterials, Nanocavities, Nanowires, Optics and photonics, Applied optics, Optical sensors, Optoelectronic devices and components

## Abstract

The controllability of photonic setups is strongly related to how coherently their outputs react to changes in their inputs; such a generic concept is treated in the case of films comprising multilayers of tilted optical axes, under visible light. The optimized designs incorporate ordinary metals or semiconductors while being able to achieve practically all the combinations of reflected, transmitted and absorbed power within the passivity limits. Importantly, most of the proposed structures exhibit substantial robustness to manufacturing defects and are fabricable with various methods. Therefore, they can make indispensable pieces of integrated photonic systems by improving their light-controlling operation for applications ranging from steering and electrodynamic switching to filtering and optical signal processing.

## Introduction

Controlling the response of photonic modules to be incorporated into integrated systems is one of the most generic objectives when designing devices that process the light and, inevitably, has involved some of the most prolific publishers of the field. Periodic dielectric materials, possessing photonic bandgaps that prohibit the propagation of photons, have been first reported to manipulate the three-dimensional vectorial electromagnetic signals^1^ while a major breakthrough towards the complete control of light in photonic crystals has been achieved by properly distributing artificial point defects^2^. Moreover, transformation optics theory has been formulated, where the exact anisotropy and inhomogeneity properties of the media are determined so that the propagation space is effectively “distorted” and full control of the magnitude and phase for the fields is achieved^3^. Importantly, customization in the group velocity of incoming beams via compact photonic circuits^4^ and all-optical switching through highly-sensitive, light-confining structures have been experimentally demonstrated at on-chip architectures^5^.

All these significant scientific progress on the control of photonic outputs via structural and textural modifications has been accompanied by sizable funding programs, encouraging the development of efficient prototypes. In particular, the US Department of Defense (DoD) has supported Multi-University Research Initiatives (MURIs) on advanced wavefront engineering and waveguiding^6^ and new phase-change materials for the integration of novel regulating devices^7^. Furthermore, National Science Foundation (NSF) has approved large-scale scientific collaborations that led to the foundation of Research Centers where media of exotic properties are used for building mobile nanomachines of tailored operation^8^ or for hosting controlled functions in inter-dimensional synthetic matter^9^. Such combined scientific and funding interest gave, naturally, rise to the fabrication of components for industrial use that offer electrically adjustable light transmission and reflection in closed spaces^10^, control of optical scattering with use of absorbing multi-coatings^11^ and electronic feedback management of the intensity noise into cavity lasers^12^.

With the advent of metasurfaces, practically every boundary condition became emulable by patterning properly engineered particles across a surface; thus, the fundamental laws of diffraction were revamped^13^ and control of electromagnetic waves has been taken to another level. More specifically, a platform with dielectric nanoposts has been formulated to provide complete control of wave polarization with subwavelength spatial resolution^14^ and simultaneously allows for full phase manipulation of the incoming light^15^. In addition, spatially dispersive metasurfaces have been designed to support arbitrary wavefront transformations^16^ while light bending and focusing of unprecedented efficacy are reported by composite metascreens^17^. Furthermore, reflectionless sheets with patches that simulate electric/magnetic currents are utilized for adjustable beam shaping^18^ and optical Huygens’ metasurfaces provide independent control of the magnitude and phase of the local reflection coefficients^19^. Note that the same metasurface concept can be successfully implemented for the manipulation of acoustic^20^ or thermal waves^21^ while light control becomes also feasible with switchable materials via mechanical stress^22^.

In this work, we consider a layered structure whose effective permittivities make a uniaxial film and the controllability concerns, in a combined way, both reflectivity and transmissivity. In particular, we define the controllability factor as the portion of all the combinations of reflected and transmitted power (under the passivity assumption) that our design can generate if excited by all possible directions and colors of the visible light. As far as the materials used for the multilayers are concerned, we regard several commonly used metals or semiconductors and optimize the configuration with respect to its volume fraction and the tilt of its optical axis; similar approaches of trying-and-testing lists from available media have been followed to determine the best crystals supporting hyperbolic light dispersion^23^ or the best bilayers for polarization engineering^24^. According to our findings, almost perfect controllability is achieved for many bulk media and with thicknesses equal to some tens of nanometers; importantly, the controllability of the obtained optimized designs does not significantly drop in the occurence of fabrication defects.

Our study serves the purpose of inverse design^25^, that recently experiences large popularity by involving novel methodology hybrids like adjoint-method gradient computations^26^, semi-analytical inversions^27^ or artificial neural networks training^28^. In addition, the considered layout can be constructed with a variety of chemical^29^ and physical^30^ techniques or via lithographic etching^31^. Therefore, the reported robust and easy-to-fabricate setups are those that exhibit the most enriched dynamics in the presence of visible light and may play important role as ultra-performing components in photonic integrated systems covering a broad range of applications from optical signal processing and imaging to beam forming and light steering.

**Figure 1:**
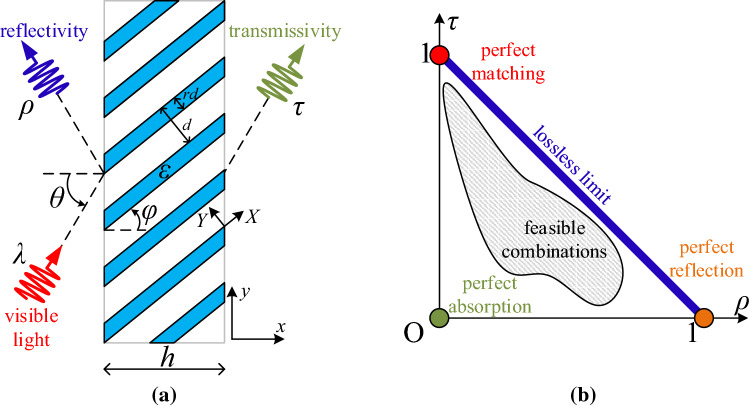
(**a**) Schematic of the proposed configuration: an obliquely incident plane wave oscillating with wavelength $\lambda$ meets under angle $\theta$ a uniaxial slab comprised of skew free-standing multilayers with tilted optical axis by angle $\varphi$. (**b**) Illustrative sketch of the controllability plane where the reflectivity $\rho$ and the transmissivity $\tau$ are represented along the two axes. The limiting cases are indicated by marker dots while the shaded region shows how controllable is the device via the incidence angle $\theta$ and the operational wavelength $\lambda$.

## Results

### Setup and metrics

We consider the photonic setup depicted in Fig. 1a, where the used coordinate system (*x*, *y*, *z*) is also defined. A plane wave of visible light with wavelength $\lambda$ meets obliquely under angle $\theta$ a slab of thickness *h*. This planar film is comprised of multilayers with period $d\ll \lambda$; each layer has size $rd<d$ and filled with a homogeneous material of relative permittivity $\varepsilon$. The multilayers are free-standing with no loss of generality and cut in such a way that their local *X* axis (shown also in Fig. 1a) forms an angle $\varphi$ with *x* direction. We assume that the incident electric field lies on *xy* plane so that the anisotropy of the structure is activated and, given the two-dimensional nature of the setup, the magnetic field is parallel to *z* axis everywhere. If the incident magnetic field is of unitary magnitude and written as: $\mathbf{H }_{inc}=\hat{\mathbf{z }}e^{-ik_0(x\cos \theta +y\sin \theta )}$, the reflective and transmissive ones take the forms: $\mathbf{H }_{ref}=\hat{\mathbf{z }}R e^{-ik_0(-x\cos \theta +y\sin \theta )}$ and $\mathbf{H }_{tran}=\hat{\mathbf{z }}T e^{-ik_0(x\cos \theta +y\sin \theta )}$ respectively, all into free space. The symbol $k_0=2\pi /\lambda$ is used for the wavenumber in vacuum and the complex reflection and transmission coefficients $\{R,T\}$ are found via the related boundary conditions.

Given the absence of higher diffractive orders^32^ ($k_0d\ll 1$), one can directly find the reflective $\rho =|R|^2$ and transmissive power $\tau =|T|^2$ into the two vacuum regions surrounding the considered slab; the absorbance is also straightforward expressed as $A=1-\rho -\tau$. In Fig. 1b, we propose a representation of the response of any two-port photonic module^33^ like the block depicted in Fig. 1a, where the horizontal axis measures the reflectivity $\rho$ while the vertical axis measures the transmissivity $\tau$. If the design is lossless, namely ${\mathrm{Im}}[\varepsilon ]=0$, then conservation of energy dictates $\rho +\tau =1$ and thus the response of the system always lies on the blue line indicating the lossless limit of Fig. 1b. Obviously, for passive films (${\mathrm{Im}}[\varepsilon ]\le 0$) the response is restricted across the lower left orthogonal and isosceles triangle of Fig. 1b; it is defined by three points describing extreme cases: perfect absorption ($\rho =\tau =0 \Rightarrow A=1$), perfect matching ($\tau =1 \Rightarrow \rho =0$) and full reflection ($\rho =1 \Rightarrow \tau =0$). As long as one changes the features of the incoming illumination, like the incidence angle $\theta$ and the oscillation wavelength $\lambda$, the response of the device gets modified accordingly and the pairs $(\rho ,\tau )$ formulate a region of feasible combinations in the parametric space of Fig. 1b. The larger is the area, the more combinations of reflectivity and transmissivity are achievable by properly selecting external excitation characteristics; thus, the portion of the orthogonal triangle occupied by sweeping $(\lambda ,\theta )$ is a metric of how controllable is the film’s response and called “controllability factor”, denoted by *CF*.

**Figure 2:**
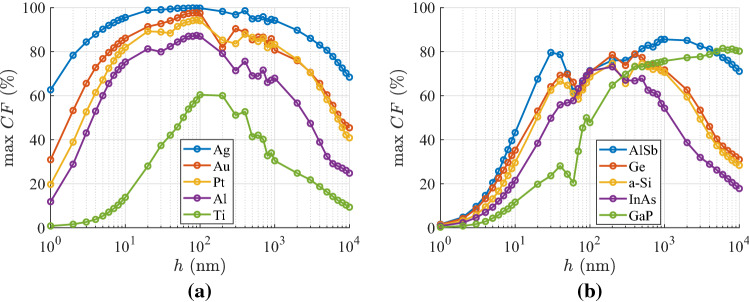
Maximal controllability factor *CF* as a function of the physical thickness of the examined film samples of Fig. 1a with respect to all tilt angles $0^{\circ }<\varphi <80^{\circ }$ and all duty cycles $0.05<r<0.95$. The material filling the multilayers is selected from a pool of several: (**a**) metals, (**b**) semiconductors.

### Maximal controllability

Our aim is to propose the parameters $(\varphi ,r)$ of thin films like this in Fig. 1a (preferably sub-wavelength *h* for the smallest wavelength $\lambda$) made of realistic media with dispersive $\varepsilon =\varepsilon (\lambda )$ that can give as many combinations of reflection and transmission (and implicitly absorption) as possible. The controllability factor *CF* will be determined by taking into account only the visible part of the wavelength spectrum ($400~\mathrm{nm}<\lambda <700~\mathrm{nm}$) and almost all possible incoming ray angles ($-\,80^{\circ }<\theta <80^{\circ }$). The computation of *CF* for a setup defined by the parametric triplet $\{\varphi ,r,h\}$ is performed numerically by thoroughly scanning the angular and frequency spectrum and represent the pairs $(\rho ,\tau )$ on a pixelated version of the map in Fig. 1b; our metric equals to the number of the occupied pixels (by one or more combinations of operational wavelengths $\lambda$ and incident directions $\theta$) over the total amount of pixels comprising the lower left triangle of Fig. 1b (passive designs). For the sake of fairness, we use exactly the same amount of pixels and the same step in sweeping $(\lambda ,\theta )$ throughout this study. A similar quantity has been already used in another context^34^ but the independent variable did not concern the incident field like in this study but the complex permittivity which, contrary to this study, was considered as a free variable.

We regard a long list of available media that can be employed in the layered layout of Fig. 1a whose simplicity allows for tight, brute force optimizations. In particular, for each material, we compute *CF* for all possible duty cycles $0<r<1$ and almost all possible tilts of the optical axis $0<\varphi <80^{\circ }$ in an attempt to find a maximum by considering arbitrary (but preferably sub-wavelength) thicknesses *h*. Note that very oblique incidences ($\theta \rightarrow \pm 90^{\circ }$) lead to trivial total reflections and thus ignored; in addition, an extremely skewed optical axis of the multilayers into the film ($\varphi \rightarrow 90^{\circ }$) makes the fabrication very challenging and therefore is excluded.

In Fig. 2a, we show the controllability performance *CF* of these optimized with respect to $(\varphi ,r)$ designs as a function of the physical size *h* of the film when the utilized materials are some of the most extensively deployed metals. One directly notices that in numerous cases the maximized *CF* surpasses the limit of $80\%$ for a properly selected *h*; remarkably, the highest controllability factors are appeared at $h\cong 90$ nm regardless of the used metal. In particular, for very thin designs ($h\rightarrow 0$), the score is low since the structure is almost transparent to the incoming illumination and the reflections are suppressed. On the other hand, for huge slabs ($h\rightarrow +\infty$), the reflection remains constant since transmission vanishes ($\tau \rightarrow 0$) as all its power is absorbed by the inevitable metallic losses; accordingly, *CF* tends to a constant low value for $h\rightarrow +\infty$. Fluctuations occurring along the curves are natural since the distance between two successive *h* values may be large while the represented function $\max _{0<r<1, 0^{\circ }<\varphi <80^{\circ }}CF$, as an outcome of optimization, is not necessarily a smooth one. By inspection of Fig. 2a, we also observe that silver-based uniaxial films exhibit the highest performance (almost $100\%$) due to the low losses of the metal, and architectures employing titanium give the poorer results due to the substantial $|{\mathrm{Im}}[\varepsilon ]|$. Indeed, large losses create significant material contrast not permitting waves to enter the sample and transmit from the other side ($\tau \ll 1$); such an effect leads to a small *CF* with combinations $(\rho ,\tau )$ far from the lossless limit of Fig. 1b.

In Fig. 2b, we repeat the same greedy optimizations in the case that the utilized materials are semiconductors and identify that the maxima of the curves appear at thicker slabs, quite different one another ($h=200{-}900$ nm). Such a feature can be attributed to the bigger, on average, $|\varepsilon (\lambda )|$ in metals and the appearance of surface plasmons if ${\mathrm{Re}}[\varepsilon ]<-1$ generating waves with arbitrarily large wavenumber that, unavoidably, shrinks the designs^35^. It should be stressed that $CF\rightarrow 0$ for tiny *h*, which is not the case in Fig. 2a, where metals, hosting the aforementioned surface effects^36^, are employed. For moderate size *h*, the worst outcome is recorded for GaP which is lossless across a substantial part of visible wavelength range; this is an intuitive conclusion since $CF\rightarrow 0$ for a lossless dielectric. Doubtlessly, its response $(\rho ,\tau )$ will be confined on the diagonal line of Fig. 1b not giving a coherent area for accessible outputs by sweeping $(\lambda ,\theta )$; that is why the losses $|{\mathrm{Im}}[\varepsilon ]|$ should be small but not zero (semiconductors or metals instead of dielectrics). It is, therefore, expected that the highest *CF* is reached for AlSb which is slightly lossy across entire the visible spectrum; mention also that its *CF* is locally maximized even for very thin structures ($h\cong 20$ nm) which is an extra evidence of increased flexibility. It is additionally noted that our metric for the GaP-based structures is maximized and takes substantial values for large thicknesses ($h>6{,}000$ nm). However, we are interested for thin films with numerous applications including visible light communication^37^; thus, we avoid sizable slabs whose much richer dynamics can obviously manipulate better the incoming signal.

**Figure 3:**
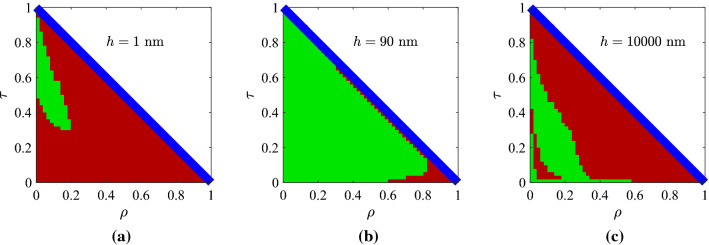
Controllability maps for the optimal Al-based designs, shown in Fig. 2a, with: (**a**) $h=1$ nm, (**b**) $h=90$ nm (optimal), (**c**) $h=10{,}000$ nm. Green pixels correspond to feasible $(\rho ,\tau )$ combinations, red to infeasible ones and white to responses attainable only via active designs (${\mathrm{Im}}[\varepsilon ]>0$). The blue line is the lossless limit of Fig. 1b.

**Figure 4:**
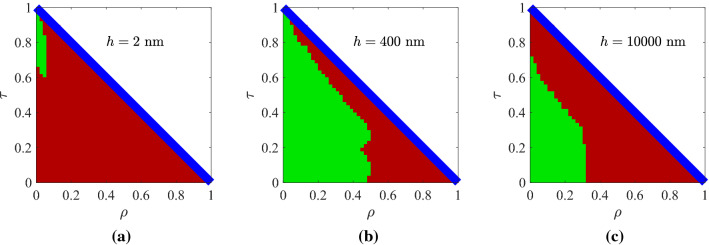
Controllability maps for the optimal Ge-based designs, shown in Fig. 2b, with: (**a**) $h=2$ nm, (**b**) $h=400$ nm (optimal), (**c**) $h=10{,}000$ nm.

In Fig. 3, we show the controllability maps concerning one of the designs of Fig. 2a (aluminum-air multilayers) for three characteristic thicknesses *h* of the sample. A green pixel indicates that this combination of reflectivity $\rho$ and transmissivity $\tau$ can constitute the response of the device for at least one incident ray with features $(\lambda ,\theta )$. A red pixel is used to label any unattainable output $(\rho ,\tau )$, the blue line shows the lossless limit of Fig. 1b, and white color corresponds to responses that require activity (${\mathrm{Im}}[\varepsilon ]>0$) to get achieved. In Fig. 3a, we consider a nanometer-thick flake of such a multilayered setup (Al-based), and we observe that large reflections cannot occur since the film is almost transparent; however, the transmissivity can be quite suppressed indicating large absorbance levels. In Fig. 3b, we demonstrate the optimal solution for Al-air multilayers for $h=90$ nm; it is remarkable that almost all combinations $(\rho ,\tau )$ are achievable, except for few of them in the corner of full reflection. Indeed, given the fact that $h\ll \lambda$, the vanishing transmission regime ($\tau \rightarrow 0$) is a challenging one; nonetheless, it can be realized for silver-based setups, as indicated by the almost flawless performance $CF\cong 100\%$ in Fig. 2a. In Fig. 3c, we consider the thickest from the examined slabs ($h=10{,}000$ nm) and we notice that the feasible response combinations get distant from the lossless limit; the moderate $|{\mathrm{Im}}[\varepsilon ]|$ of aluminum with the large *h*, creates substantial ohmic losses and the result would be close to the *CF* of a half-space. Interestingly, a hole into the green pixelated region is recorded and means that the mapping of a coherent $(\lambda ,\theta )$ area into $(\rho ,\tau )$ plane may be non-conformal, for specific thicknesses, due to the developed phase mismatch.

In Fig. 4, we regard a representative design deploying germanium, a commonly used semiconductor. In Fig. 4a, we investigate a thin slab of $h=2$ nm and notice the poor *CF* score comprising responses around the perfect matching regime ($\rho \rightarrow 0$); we avoid to examine the case of $h=1$ nm which gives a $CF\cong 0$, as shown in Fig. 2b. Once again, we understand the advantage of metallic (${\mathrm{Re}}[\varepsilon ]<0$) designs against the semiconducting (${\mathrm{Re}}[\varepsilon ]>0$) ones; doubtlessly, the Al-based film of Fig. 3a is thinner than the Ge-based of Fig. 4a but of higher *CF*. In Fig. 4b, we consider the optimal thickness for that type of multilayers and notice a substantial *CF*, though much less than that of Fig. 3b, avoiding many responses close to lossless limit and around the full reflection. In Fig. 4c, we increase the size of the sample to $h=10{,}000$ nm and, unlike in Fig. 3c, the drop in the performance is milder, and the domain of feasible combinations is characterized by coherence.

**Figure 5:**
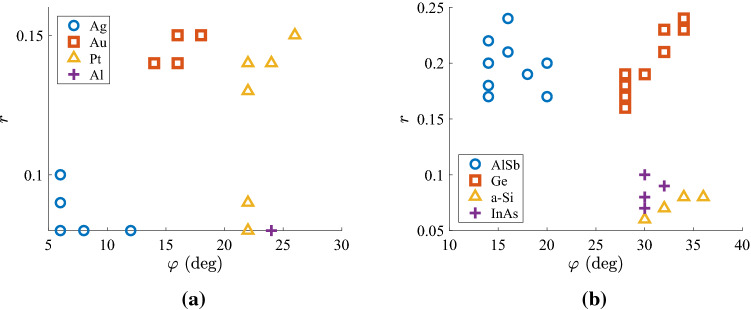
Optimal structural parameters (tilt of optical axes $\varphi$, filling factors *r*) when the sample thickness *h* deviates around its optimized value, found in Fig. 2, by $\pm 10\%$ when: (**a**) metal are incorporated, (**b**) semiconductors are incorporated.

### Optimal films

It would be interesting to understand what is the preference of the optimal designs shown in Fig. 2 in terms of the materials analogy *r* and the tilt $\varphi$ in the multilayers. In Fig. 5, we focus on that thicknesses *h* giving the highest scores for each material used and we represent the optimal values on the map $(\varphi ,r)$ of the structural parameters if one perturbs slightly ($\pm 10\%$) that optimal size *h*. In Fig. 5a, we regard the four more successful cases incorporating metals and report small deviations in the optimized $(\varphi ,r)$, especially when it comes to plasmonic portion *r*. Furthermore, we notice that Al-based designs exhibit substantial insensitivity with respect to *h* while insisting to tiny duty cycles *r* and significant tilts $\varphi$; on the contrary, Pt-air multilayers modify substantially their optimal metallic portion *r* for a moderate change in the size *h*. It is also remarkable that the champion silver-based setups favor very slight tilts $\varphi$ and small duty cycles *r*. In Fig. 5b, we examine semiconductor-based films and repeat the process of Fig. 5a; we observe considerable dispersion around the mean values which is natural since the thicknesses *h* are larger. Again, the highest-performing film (with AlSb) demands moderate angles $\varphi$ but much larger textural analogies *r*, compared to Fig. 5a. Oppositely, the rest of the samples require increased tilts $\varphi$ mostly independent from *h*; two of them (with a-Si and InAs), are least prone to *h*-changes and prefer low duty cycles *r*.

**Figure 6:**
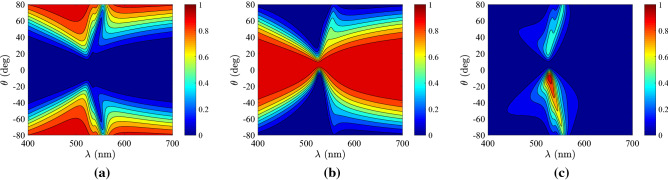
Response of the optimal Ag-based design, according to Fig. 2a, with respect to the oscillating wavelength $\lambda$ and the incidence angle $\theta$. (**a**) The reflectivity $\rho =\rho (\lambda ,\theta )$, (**b**) the transmissivity $\tau =\tau (\lambda ,\theta )$, (**c**) the absorbance $A=1-\rho -\tau$. Plot parameters: $h=90$ nm, $\varphi =6^{\circ }$, $r=0.08$.

It is meaningful to investigate the response of optimal samples both in frequency and angular spectrum in order to understand how the combined levels for reflectivity and transmissivity are achieved. In Fig. 6, we analyze the highest-performing Ag-based design and represent the reflectivity $\rho$, the transmissivity $\tau$, and the absorption $A=1-\rho -\tau$ as functions of the oscillating wavelength $\lambda$ and the incidence angle $\theta$. In Fig. 6a, we notice that reflection is small (almost zero) for directions $\theta$ close to normal and gets more substantial (almost $100\%$) as one approaches the grazing angle ($\theta =90^{\circ }$). Note that $\rho$ is small for violet and red color and around $\lambda \cong 550$ nm, abrupt switches are occurred. In Fig. 6b, the transmissivity exhibits an almost complimentary to $\rho =\rho (\lambda ,\theta )$ variation since it is high across a large angular sector centralized at $\theta =0$, with the exception of a narrow-angle selectivity at $\lambda \cong 550$ nm; all values for $\rho$ are accessible. However, the patterns are not perfectly symmetric and thus the sum of the two quantities $(\rho +\tau )$ is not equal to unity, due to the presence of losses; indeed, in Fig. 6c the absorption is maximized across two asymmetric parametric “islands” in the vicinity of $\lambda \cong 550$ nm, the strongest of which appears for $\theta <0$; note that even perfect absorption is feasible for a specific pair of $(\lambda ,\theta )$.

**Figure 7:**
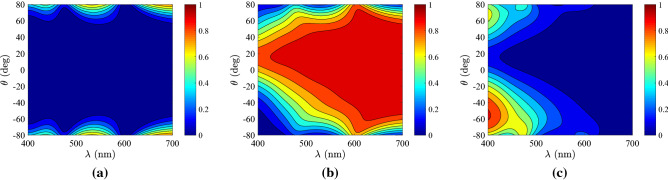
Response of the optimal AlSb-based design, according to Fig. 2b, with respect to the oscillating wavelength $\lambda$ and the incidence angle $\theta$. (**a**) The reflectivity $\rho =\rho (\lambda ,\theta )$, (**b**) the transmissivity $\tau =\tau (\lambda ,\theta )$, (**c**) the absorbance $A=1-\rho -\tau$. Plot parameters: $h=900$ nm, $\varphi =14^{\circ }$, $r=0.22$.

In Fig. 7, we repeat the calculations of Fig. 6 but for the highly-controllable AlSb-based design. In Fig. 7a, where the variation of $\rho =\rho (\lambda ,\theta )$ is depicted, we notice that reflections are very low across the entire parametric box apart from some increase when angle of incidence becomes very oblique. Note that the response is far from the full reflection regime ($\rho \rightarrow 1$), as indicated by Fig. 4b (similar outcome of the Ge-based structure). In Fig. 7b, we show the transmissivity $\tau =\tau (\lambda ,\theta )$, which is substantial at most directions and wavelengths but covers almost the entire range $0<\tau <1$; it again possesses an asymmetric pattern as imposed by tilt of optical axis $\varphi$ of the multilayers. In Fig. 7c, we represent the absorption which, by suitably selecting the incoming illumination, can obtain all values from vanishing to close to unity; it seems that the maximal $A=1$ may be taken outside of the consider frequency band at the ultra-violet part of the spectrum.

### Designs robustness

Even at the frequencies of visible light, multilayered structures like the one depicted in Fig. 1a are possible to get fabricated via a variety of construction techniques. Chemical Vapor Deposition (CVD) is a major category of them that implements chemical reactions along the surfaces allowing for ultra fine thickness control in layer-by-layer development. In the same way, Atomic Layer Deposition (ALD) is capable of producing thin multilayers from a variety of materials established on sequential, self-limiting reactions^29^; it is successfully used in creating structures with a high permittivity contrast^38^, exactly as ours. Furthermore, Physical Vapor Deposition (PVD) approaches like evaporation of metals that diffuses to a substrate or, alternatively, sputtering that requires generation of plasma and strikes of ions forming additional layers. Importantly, Molecular Beam Epitaxy (MBE) is one of the most popular and precise physical methods where the required texture is grown molecule-by-molecule via shooting from effusion cells^30^, exceptionally befitted to the manufacturing of heterostructures consisting of stacked monolayers^39^. Even self-assembling routes towards multiple parallel lamellar inclusions have been reported with applications in tunable narrow-band optical filters^40^ and energy accumulators^41^. As far as the precise cutting of the thin samples is concerned, it can be performed by utilizing Electron Beam Lithography (EBL) followed by lift-off processes^31^ admitting the transfer to rigid and flexible substrates^42^.

**Figure 8:**
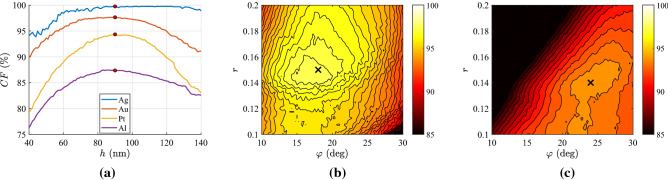
Robustness of the optimal designs incorporating metals. (**a**) Controllability factor *CF* as a function of the physical thickness *h*—the optimal regimes are denoted by red dots. (**b**) Variation of controllability factor *CF* with respect to optical axis tilt $\varphi$ and the duty cycle *r* for gold-based multilayers ($CF\cong 98\%$)—the optimal regimes are denoted by black $\times$. (**c**) Same as (**b**) for platinum-based multilayers ($CF\cong 94\%$).

However, finesse in the fabrication should not be taken for granted and, accordingly, it is important to examine the sensitivity of the performance *CF* with respect to changes in the thickness *h* of the sample, defects in the tilt angle $\varphi$ and imperfections in the materials portion *r*. In Fig. 8a, we show the variation of *CF* for several metal-based setups as a function of the size *h* with fixed $(\varphi ,r)$ at the optimal values, while the corresponding thickness is marked by a red dot. First of all, the shape of the curves is somehow shaky due to the way we evaluate *CF* based on discrete pixels. In addition, we notice that a thicker structure with identical characteristics does a better job in terms of controllability compared to a thinner one. The least robust design is the one made of platinum, while the most resilient is the one with Ag-air multilayers. Gold-based setup exhibits a remarkable stability with respect to the slab’s size and aluminium-based layout delivers poorly if the film gets cut thinner by mistake.

In Fig. 8b, we pick one of the setups analyzed in Fig. 8a (gold-air design), and this time we perturb the parameters $(\varphi ,r)$ of the multilayers by keeping the optimal thickness *h* constant; the ideal operational regime is marked by a black $\times$. Despite the substantial change in the stack geometry, the performance of the devices does not drop significantly except for a combined increase in the tilt $\varphi$ and a strong decline in the duty cycle *r*. In Fig. 8c, a less successful design (platinum-air multilayers) is tested with respect to changes in $(\varphi ,r)$. Again, high robustness is recorded but the situation worsens clearly for the opposite factors to those of Fig. 8b (smaller $\varphi$, higher *r*).

**Figure 9:**
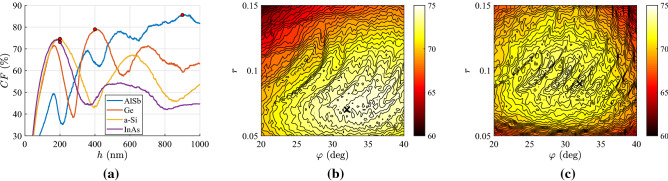
Same as Fig. 8 but for semiconductor-based designs. (**a**) Controllability factor *CF* as a function of the physical thickness *h*—the optimal regimes are denoted by red dots. (**b**) Variation of controllability factor *CF* with respect to optical axis tilt $\varphi$ and the duty cycle *r* for amorphous-silicon-based multilayers ($CF\cong 75\%$)—the optimal regimes are denoted by black $\times$. (**c**) Same as (**b**) for InAs-based multilayers ($CF\cong 73\%$).

In Fig. 9, we repeat the calculations of Fig. 8 but for the various semiconductors investigated in Fig. 2b. In Fig. 9a, we depict our metric *CF* across a much more extended range of thicknesses *h* and we realize that even non-plasmonic designs are quite robust. Especially AlSb-based film retains the same performance for over one micron around its working value ($h=900$ nm); on the contrary, the rest of designs experience a drop of their *CF* within some tens of microns from their operational point simply because their optimal thicknesses are much smaller ($h=100{-}200$ nm). In Fig. 9b, we examine the silicon-based setup and we again notice the noise-like behavior like in Figs. 8 and 9a; importantly, the deterioration in *CF* is much milder compared to the plasmonic multilayers but the average performance smaller. Similar conclusions are drawn from Fig. 9c where the response of the optimal InAs-based design is examined; oppositely to Fig. 9b, the controllability factor is harmed more by larger tilts $\varphi$ and smaller analogies *r*. In any case, it is clear from Figs. 8 and 9 that the proposed films, regardless of the used medium, are not significantly affected by imperfections in thickness, multilayer growing and sample cutting.

## Discussion and conclusions

Discovering structures that exhibit maximally rich electrodynamics is directly associated with the controllability of their photonic response. In this study, we have defined controllability by evaluating the variation of the output, once the input is being swept across direction and frequency; if that output changes substantially, then the corresponding device can be characterized as highly controllable, otherwise not. This concept has been applied to films comprising multilayers, separated by airgaps, with tilted optical axis and illuminated by visible light; at each thickness of the slabs, we optimize the aforementioned controllability factor with respect to the density of the stacked layers and the tilt.

We remarkably show that such a simple setup, in case it is excited by a wide spectrum of angles and colors, can respond in all the possible combinations of reflection, transmission and absorption. By considering several metals and semiconductors as the filling materials of the stacks, it is found that the optimal size of the film should not be neither too small, where the sample is practically transparent, nor too big so that the transmissivity is not blocked. As usually happens in several photonic effects, the maximal performance is recorded for low-loss plasmonics (like silver), where the developed surface waves are admitted to effectively shrink the physical dimensions and offer significant operational flexibility to the hosting designs. The reported films are additionally quite robust to fabrication defects such as imperfect thicknesses, miscalculation of tilt angles or overestimation of materials duty cycles.

Such optimal films that possess so extensive a repertoire of responses under visible light, can be extremely useful as components in integrated photonic systems. Indeed, most photonic devices that create, manipulate and detect optical signals require controlling parts that modify their outputs in a maximal way as their inputs are changing. Therefore, this study may inspire experimental efforts towards the fabrication, testing and packaging of the proposed setups so that they will serve well a variety of roles from switching and filtering to beam steering and imaging.

## Methods

Since the multilayered structure of Fig. 1a has a spatial period *d* much smaller than the oscillation wavelength $\lambda$, we can replace it by its homogenized analogue^43^. The effective permittivies along their major axes (*X*, *Y*) are given by: $\varepsilon _X=(1-r)+\varepsilon r$ and $\varepsilon _Y=\frac{\varepsilon }{(1-r)\varepsilon +r}$ and thus the permittivity tensor $[\varepsilon ]$ in the global Cartesian coordinate system (*x*, *y*, *z*) takes the form^44^:

1 $$\begin{aligned} {[}\varepsilon ]=\left[ \begin{array}{ccc} \varepsilon _{xx} &{}\quad \varepsilon _{xy} &{}\quad 0 \\ \varepsilon _{xy} &{}\quad \varepsilon _{yy} &{}\quad 0 \\ 0 &{}\quad 0 &{}\quad \varepsilon _X \end{array}\right] =\left[ \begin{array}{ccc} \varepsilon _X\cos ^2\varphi +\varepsilon _Y\sin ^2\varphi &{}\quad (\varepsilon _X-\varepsilon _Y)\cos \varphi \sin \varphi &{}\quad 0 \\ (\varepsilon _X-\varepsilon _Y)\cos \varphi \sin \varphi &{}\quad \varepsilon _Y\cos ^2\varphi +\varepsilon _X\sin ^2\varphi &{}\quad 0 \\ 0 &{}\quad 0 &{}\quad \varepsilon _X \end{array}\right] . \end{aligned}$$

It should be noticed that the width *h* does not significantly affect the permittivity homogenization because it is related to the assumption for an optically small spatial period of the multilayers ($k_0d\ll 1$). However, we advocate that such an approximation gets more successful for an increasing *h*, since the edge effects due to the finite length of layers weaken. Since most of our optimal designs possess a thickness $h>30$ nm and *d*, via modern fabrication methods, can be downsized to a few nanometers, the approximate formulas (1) may capture well the dynamics of the wave interactions with the structure.

The incident, reflective and transmissive magnetic fields are written as: $\mathbf{H }_{inc}=\hat{\mathbf{z }}e^{-ik_0(x\cos \theta +y\sin \theta )}$, $\mathbf{H }_{ref}=\hat{\mathbf{z }}R e^{-ik_0(-x\cos \theta +y\sin \theta )}$ and $\mathbf{H }_{tran}=\hat{\mathbf{z }}T e^{-ik_0(x\cos \theta +y\sin \theta )}$ respectively, where $k_0=2\pi /\lambda$ is the wavelength into free space. The reflection and transmission coefficients $\{R,T\}$ are determined as follows, by imposing the necessary boundary conditions at the interfaces between air and the film.

2 $$\begin{aligned} R&= \frac{2i\sin (kh)(u^2-k^2\varepsilon _{xx}^2)}{e^{+ikh}(u + k \varepsilon _{xx})^2-e^{-ikh}(u - k \varepsilon _{xx})^2}, \end{aligned}$$

3 $$\begin{aligned} T&= \frac{4e^{ik_0h\left( \cos \theta +\frac{\varepsilon _{xy}}{\varepsilon _{xx}}\sin \theta \right) }u k \varepsilon _{xx}}{e^{+ikh}(u + k \varepsilon _{xx})^2-e^{-ikh}(u - k \varepsilon _{xx})^2}, \end{aligned}$$

where $u=k_0(\varepsilon _{xx}\varepsilon _{yy}-\varepsilon _{xy}^2)\cos \theta$ and *k* is given by:

4 $$\begin{aligned} k=k_0\sqrt{(\varepsilon _{xx}-\sin ^2\theta )\left( \frac{\varepsilon _{yy}}{\varepsilon _{xx}}-\frac{\varepsilon _{xy}^2}{\varepsilon _{xx}^2}\right) }. \end{aligned}$$
